# The strength of our stories: a qualitative analysis of a multi-institutional GME storytelling event

**DOI:** 10.1080/10872981.2021.1929798

**Published:** 2021-06-07

**Authors:** Maren E. Olson, M. Lynne Smith, Alexandra Muhar, Trisha K. Paul, Bernard E. Trappey

**Affiliations:** a Department of Medical Education, Children’s Minnesota; Department of Pediatrics, Center for the Art of Medicine, University of Minnesota, Minneapolis, USA; b Department of Educational Foundations, College of Education, Criminal Justice, and Human Services, University of Cincinnati, Cincinnati, USA; c Department of Pediatrics, Division of Neonatology, Vanderbilt University, Nashville, USA; d University of Minnesota.; e Departments of Internal Medicine and Pediatrics, Center for the Art of Medicine, University of Minnesota, USA

**Keywords:** Storytelling, narrative medicine, graduate medical education, medical humanities, resilience, burnout, Humanities

## Abstract

**Context**: Storytelling is a powerful tool for encouraging reflection and connection among both speakers and listeners. While growing in popularity, studying the benefits of formal oral storytelling events within graduate medical education remains rare. Our research question was: could an oral storytelling event for GME trainees and faculty be an effective approach for promoting well-being and resilience among participants?

**Methods**: We used multiple approaches to gather perspectives from physician participants (storytellers and audience members) at an annual oral storytelling event for residents, fellows, and faculty from seven academic health systems in Minnesota. Data sources included short reflections written by participants during the event, an immediate post-event survey exploring participants’ experiences during the event, social media postings, and targeted follow-up interviews further exploring the themes of connection and burnout that were raised in post-event survey responses. We performed a qualitative analysis using both deductive and inductive coding to identify themes.

**Results**: There were 334 participants, including 197 physicians. At the event, 129 real-time written reflections were collected. There were also 33 Twitter posts related to the event. Response rate for the post-event survey was 65% for physicians, with 63% of physician respondents volunteering for targeted follow-up interviews. Of those, 38% completed the follow-up interview. Themes that emerged from the multi-modal qualitative analysis included a sense of connection and community, re-connection with meaning and purpose in work, renewal and hope, gratitude, and potential impact on burnout.

**Conclusion**: The large turnout and themes identified show how an oral storytelling event can be a powerful tool to build community in graduate medical education. Qualitative analysis from multiple sources obtained both in real-time at the event and upon deeper reflection afterwards showed the event positively impacted the well-being of participants and that oral storytelling events can be an effective approach for promoting resilience in GME.

## Introduction

*I still think about some of the stories I heard and the emotions that were shared. I have had so many instances in my own training where I felt helpless, that I was a bad physician, or that I had a hard time leaving behind emotional experiences I have had at work, and I think that the Story Slam reminded me that I was not alone with my feelings. It is an encouragement to continue to share our stories because you never know how it might resonate with someone else*. -resident

Storytelling has resurged in popularity in recent years. *The Moth*, a non-profit organization ‘dedicated to the art and craft of storytelling,’ sponsors live storytelling events known as ‘story slams’ and produces a popular National Public Radio show [1]. An understanding of the importance of sharing stories has surfaced in medicine as well, with advocates of a field known as Narrative Medicine reminding us that the telling and understanding of stories lie at the roots of the patient-physician relationship [2]. Oral storytelling is also gaining traction among physicians, as evidenced by the popularity of *The Nocturnists* medical storytelling podcast [3]. However, there is a paucity of research on the use of oral storytelling in graduate medical education (GME).

Stories matter. For millennia [4,5], humans have told stories to make sense of the world, articulate shared understanding, and build community. Medicine is no exception. We use stories in our everyday work as we describe the salient features of a disease process, build rapport with our patients, and teach trainees about the culture of our field.

Stories also have potential to heal. Through stories, we share our experiences, find meaning in the midst of grief and loss, and buoy each other through challenging times. In an era when so many in medicine struggle with burnout [6–14], sharing stories also has the potential to build resilience, which protects against burnout [15–17]. Feeling connected with others and having a sense of purpose or meaning about work are associated with increased resilience and decreased burnout in physicians [17–19]. In addition, adequately processing traumatic events promotes healing [20,21].

Given this knowledge, we had previously undertaken a pilot project in 2018 involving a live, community-wide storytelling event (Story Slam) as a strategy for proactively impacting well-being among residents and fellows in the Minneapolis-St. Paul metro area in the hopes of promoting of a sense of community, encouraging physicians to reflect on what makes their work meaningful, and to help them process challenging experiences. The event featured residents, fellows, and faculty sharing 750-word stories they had prepared in advance. We demonstrated that the approach was both feasible and well-accepted [22].

As the recent AAMC report, *The Fundamental Role of the Arts and Humanities in Medical Education* highlights, there is a paucity of research of the benefits of incorporating the arts and humanities into medical education [23]. This is certainly true regarding the potential impact of oral storytelling events in graduated medical education on well-being and resilience. Indeed, we are aware of only a single small study related to this topic [24]. Therefore, given the success of the inaugural Story Slam described above, we planned a 2^nd^ annual Story Slam event and the research study we will describe here, in order to begin addressing this gap.

### Theoretical framework

Reflective writing – a form of reflective practice [25] – has been shown to improve health and well-being in the general population [20,26]. Reflective writing is thought to improve wellbeing through the organization of thought into a coherent narrative, allowing the writer to find meaning and purpose in their experiences [27,28]. Additionally, oral storytelling can build community and physicians who feel more connected to those around them are more resilient and thus at less risk for burnout [4,15,19,29–31].

Using a social constructivism framework [25,32,33], we postulated that reflective writing has the potential to benefit others when that writing is shared as an oral story. Social constructivism states that learning and the construction of meaning occurs when individuals are engaged in social activities such as interaction and collaboration, particularly through the use of language [34]. During oral storytelling, the brain activity of storytellers (who have previously engaged in reflective practice through the composition of the stories that they perform) and listeners align through the phenomenon of neural coupling [35,36]. Furthermore, this coupling is enhanced when the stories told and heard are emotionally complex [37]. Thus, audience members vicariously experience the emotions of the storyteller and are prompted to reflect on similar experiences that they may have had in their own careers, building a sense of shared feeling and connection. This experience of community has the potential to be protective: physicians who feel more connected to those around them are more resilient and thus at less risk for burnout [4,15,19,29–31].

Therefore, we designed this study to explore the experiences of physician-storytellers and physician audience members (henceforth collectively referred to as ‘participants’) during the 2^nd^ annual storytelling event for residents and fellows in the Minneapolis-St. Paul metro area, referred to henceforth as the ‘Story Slam.’

Our research question was: could an oral storytelling event for GME trainees and faculty be an effective approach for promoting well-being and resilience among participants?

## Methods

A planning committee with faculty and residents representing multiple institutions organized the Story Slam. A call was made for 750 word story submissions, with no specific theme identified. The event was publicized via GME listservs, social media, posters, and word-of-mouth. A target number of 15 stories was set by the planning committee, and stories were accepted in the order received, with the caveat that trainee submissions took precedence over faculty entries. This free event was held on a weekday evening at a popular local brewery.

### Participants

The Story Slam was open to residents, fellows, and faculty from seven academic institutions in the Minneapolis/St. Paul, Minnesota metropolitan region. Together, these institutions train over 1300 residents and fellows in more than 140 GME programs. All of these trainees were invited to the event. Significant others were welcome to attend as well.

### Philosophical framework

The philosophical framework guiding the data collection and analysis was phenomenology, because we wanted to learn how participants interpreted the experience of the story slam and what meanings they attribute to that shared experience. Phenomenology involves focusing on the experience that a study’s informants have, and depicting, for a study’s readers, the essence of that experience.

### Data collection

During the Story Slam, we invited participants and their guests to reflect upon their experience. Sticky notes were available on each table with the prompt, ‘*We invite you to use the sticky notes on your table to jot down any thoughts or emotions that come to mind as you listen to tonight’s stories.’* People were given the option to keep their reflections private or allow them to be collected over the course of the evening. Collected reflections were displayed (please see Supplemental Digital Appendix 1) and people had the opportunity to add additional reflections to the display.

At the close of the event, we invited those in attendance to use Twitter to post about the Story Slam using a specific hashtag. (That hashtag is not included in this manuscript to protect posters’ privacy.) The same hashtag was used in publicity preceding the event and posted prominently in the event space. Several days after the event, we performed a search on Twitter using the designated hashtag; tweets using it were de-identified and entered into a secure database for analysis.

Participants and their guests were asked to complete a brief post-event Qualtrics-based survey about their experience at the event (please see Supplemental Digital Appendix 2). QR codes for the survey were on event programs and posted around the venue. We also sent the survey to attendees the day after the event, asking those who had not already completed it to please respond. The survey closed with a request for participants willing to participate in a brief interview about their experience at the Story Slam. Questions for the interviews were generated based on the information gathered in the follow-up survey and during our initial analysis of the written comments. De-identified email and text communications about the event sent to event organizers by participants were also included as an additional source of data.

Given an unexpectedly large number of participants willing to do follow-up interviews and the complexity of physicians’ schedules, we opted to email the follow-up questions to participants (please see Supplemental Digital Appendix 3). The follow-up questions were sent to participants one week after the event. They were invited to respond to the questions via email, submit a recording of their responses, or via phone interview with a research team member. Responses were collected over the course of the next 2.5 weeks and were transcribed, de-identified, and entered into a secure database for analysis.

### Data analysis

Likert scale questions from the post-event survey were converted to a 1 to 5 scale and mean responses for all respondents were calculated for each question. Narrative responses for each of the open-ended questions, ‘*Please describe your experience at tonight’s event’* and *‘Any other comments or suggestions?’* from the post-event surveys were compiled. For follow-up questions, responses were de-identified by a member of our research team [AM] who did not participate in analysis of the in-depth follow up data, in order to ensure confidentiality. Post-event survey and follow-up responses were categorized based on the role of the respondent (attending/fellow/resident/other).

Non-physician responses to the post-event survey (i.e., anyone who marked ‘other’) were excluded from analysis and non-physicians were not invited to participate in an interview; their perspectives are beyond the scope of this study. While it was not possible to determine whether sticky-note responses or Twitter posts were from physicians or non-physicians, the research team opted to include these data sources nevertheless because they captured real-time emotion in a way no other data sources did and also allowed for additional data triangulation.

MO and BT did preliminary coding of the post-event responses and generated additional questions to address with the in-depth follow up interviews. Coding was done using both deductive and inductive methods, with our research question and existing literature helping identify some of the codes. Sentences/phrases from post-event surveys, in-depth follow-up, sticky notes and tweets were coded with the option to assign multiple codes to each item. Each member of the research team first coded independently. Then, MO reviewed each list of codes, grouped related codes into themes using both frequency of repetition within the data, our research question, and our conceptual framework. MO, BT, and LS met multiple times to refine codes and discuss emerging themes. All members of the research team agreed upon final themes [38].

Multiple techniques were employed to increase the trustworthiness of our study [39]. First, we have increased the credibility of our results using data triangulation, specifically by gathering data during and immediately after the event, as well as in the several weeks following it [39]. Collected data included: fieldnotes recorded during the event by the primary investigator; post-event, Qualtrics-based surveys completed by both storytellers and audience members; Twitter postings; post-it note reflections written during the story slam; and e-mail/text correspondence about the event between participants and research team members in the weeks after the story slam. Second, analyst triangulation was utilized to increase the rigor of the data analysis. Multiple members of the research team coded the collected data independently, then met to reach agreement on codes, recoded the data independently, then met again to agree upon themes in the coded data and direct quotes from the data representative of those themes. Third, to increase transferability [39] we used rich, thick description [40] in our write-up of the results. Finally, dependability and confirmability have been ensured through the use of an audit trail detailing the research methods used and decision points encountered during the study.

Privacy and ethical concerns were addressed in several ways. Survey responses were anonymous. Names and contact information for in-depth follow-up participants could not be linked to post-event survey responses. Identifying information was removed from survey and interview responses, Twitter posts, and texts.

Ethical review was done by the Children’s Minnesota Institutional Review Board (IRB) and they determined this study was exempt.

## Results

Participants consisted of 132 residents, 14 fellows, 51 faculty, and their guests, for total of 343 people. This included 15 storytellers (12 residents, 1 fellow, 2 faculty) who shared poignant, hope-filled, and sometimes gut-wrenching stories about their experiences in medicine. Each story lasted about 5 minutes and the overall event was approximately 2 hours in length. For examples of the types of stories told, we recommend reading two that were subsequently published [41,42]. There were 129 participants who completed the post-event survey (81 residents, 10 fellows, 38 faculty) and 31 participants who completed the in-depth follow-up questions (22 residents, 2 fellows, 7 faculty). Participants’ response to the event was overwhelmingly positive. For the question, *How likely would you be to recommend a future Story Slam to friends and colleagues*?, the mean response was 4.8 out of 5, with 1 being *Not at all likely* and 5 being *Extremely likely*. Qualitative analysis of the data collected revealed five main themes: an experience of connection and community, a sense of meaning and purpose, feelings of renewal and hope, gratitude, and potential impact on burnout. All themes were evident among the various study participants and data sources. Descriptive statistics about data collected are outlined in Table 1.

**Table 1.** Descriptive statistics regarding event participants and study data

Event Participants	
Total # of participants	343
Physicians (all)	197
Residents	132 (67%)
Fellows	14 (7%)
Faculty	51 (26%)
Guests of above physicians	146
**Post-event survey**	
Physicians (all)	129 responses (65% response rate)
Residents	63% (n = 81)
Fellows	8% (n = 10)
Faculty	29% (n = 38)
**In-depth follow-up**	
Total # of volunteers	81 (63% of survey respondents)
Completed follow-up	31 (38% of volunteers))
Email response	28 (90%)
Audio file response	2 (7%)
Phone interview	1 (3%)
**Additional data sources**	
Sticky note reflections^a^	129
Twitter posts^a, b^	33
Posts with comments	12 (36%)
Re-posted Tweets	16 (48%)

^a^Sticky note reflections and Twitter posts may be from either physicians or non-physicians. Please see Methods section for further explanation

^b^Some posts were by members of the research team who happen to be Twitter users. The posts were in line with their typical use of Twitter and not intentionally informed by this research project; none of those posts were included in this paper.

### Connection and community

Across data sources, participants consistently mentioned a sense of connection and community as a powerful aspect of participating in the Story Slam. One resident wrote, *‘This event was amazing – to gather with residents from across the Twin Cities and feel a part of such a fantastic community was a blessing ….’* [survey, resident #4] Both storytellers and audience members described how the event helped them feel connected to others working in medicine:

*I felt a better sense of community after leaving the event and I was reminded that my colleague community goes beyond my residency class and the attendings and fellows I am currently working with and extends to other disciplines and specialties that I don’t personally interact with as often. I think this event was a lovely reminder of the greater medical community support system we have …* . [follow-up, resident #3]

Moreover, among participants who completed the follow-up questions, many believed that the connection that was fostered would endure, especially if reinforced.

*I think it will persist. It’s a powerful thing to have a human connection to the storyteller, especially about a topic that may be very similar to an experience of your own*. [follow-up, resident #5]

Of note, the theme of connection and community was more prevalent in the data than any other theme. Please see Table 2 for examples of quotes demonstrating this theme.

**Table 2.** Sample quotes for the themes *Connection and Community, Meaning and Purpose, Renewal and Hope*, and *Gratitude.*

Theme	Example Quotes
**Connection and Community**	*I felt connected to people in a way I don’t in my clinical practice. I felt accepted, represented, and thankful for the vulnerability*. [survey, faculty #30] *I felt a sense of community and connection from this event*. [survey, resident #68] *Connected me back to my own experiences in training, to my feelings of connection and empathy for current trainees …* . [survey, faculty #16] *The [power] of stories. They connect us/normalize our common humanity. We laughed, cried and belonged tonight regardless of age, sex, gender, color*. [Twitter] *I think the sense of connection started at Story Slam [sic]will continue if we make a concerted effort to share our stories of struggle and success with our colleagues*. [follow-up, faculty#3]
**Meaning and Purpose**	*Very meaningful experience. I think the shared vulnerability made us all stronger*. [survey, fellow #10 (storyteller)] *What an incredible event. A safe space to come share what is beautiful and difficult and hilarious about our jobs and training is therapeutic for the medical community*. [survey, faculty #9] *Incredibly meaningful to share our thoughts and process emotions with supportive colleagues who have gone through the challenges that make up residency*. [survey, resident #81 (storyteller)] *I have definitely been reflecting more on my experiences in medicine and their impact on me and others after the event. The people who shared really moved me and helped to reinvigorate my love for this practice*. [follow-up, faculty #5] *This event made me reflect on my own experiences I have had as a resident as well as how these experiences can bind us together as a medical community. Before the Story Slam, I hadn’t realized how much my own stories were weighing on my heart and mind every day*. [follow-up, resident #13]
**Renewal and Hope**	*Amazing stories. Made me laugh and cry. Renewed my faith in Medicine. Gave me the will to continue on*. [survey, resident #12] *We have common themes that haunt, encourage, and sustain us. Sharing tempers the sorrow and expands the joy. I’m replenished*. [survey, faculty #37] *My heart is full*. [sticky note response] *This filled my tank*. [sticky note response]
**Gratitude**	*Grateful for the opportunity to reflect on medical training and share with an incredibly supportive community!* [Twitter] *Extremely grateful to be around such thoughtful and genuine people*. [survey, resident #45] *Thank you to all the learners whom I am supposed to be teaching, for teaching me how to return to myself*. [survey, faculty #2] *Thank you! We want more of this!* [survey, resident #47] *The story slam was outstanding. Thank you for all the work that went into bringing together residents and helping us connect. Just awesome. ❤* [resident, text message]

^a^Sticky note reflections and Twitter posts may be from either physicians or non-physicians. Please see Methods section for further explanation

### Meaning and purpose

Many participants discussed how the Story Slam helped them re-connect with a sense of meaning and purpose in their work. One resident described, *‘I feel so inspired to keep on going … even on the days when I really want to slow down.’* [survey, resident #38]. A faculty member wrote, *‘It was a very affirming experience. It reminded me why I went into medicine.’* [survey, faculty #13]

Participants also shared how the event prompted them to reflect upon their own experiences, and how that, in turn, was meaningful.

*When I go through my own experiences, it’s hard to remember those are “stories,” because it just feels like everyday life while I’m in the midst of them. But after a gathering like this, where I could hear the impact of other people’s stories, I realized how many of my own stories I have. After almost every story that was shared, I found myself thinking “I’ve experienced that too!” It was helpful to see the non-resident attendees react to the stories that were shared, because that made me believe that my stories could also be impactful, and they’re worth sharing too*. [follow-up, resident #7]

As evidenced by these quotes, the experience of the Story Slam served to remind participants about why they chose a career in medicine and how that work is meaningful to them. Please see Table 2 for examples of quotes demonstrating the theme *Meaning and Purpose*.

### Renewal and hope

Participants described feeling renewed by the Story Slam. They reported being more hopeful and empowered to continue their work (Table 2). One resident wrote, *‘Amazing stories. Made me laugh and cry. Renewed my faith in Medicine. Gave me the will to continue on.’* [survey, resident #12]. Faculty expressed similar sentiments, ‘*This event restores my soul after a very tough week on service at the hospital’* [survey, faculty #2] The theme was also present in sticky note responses, such as *‘Wow. I feel good about the future of medicine.’*

### Gratitude

Gratitude was another theme throughout the data sources (Table 2). Some participants expressed gratitude for specific stories, such as a sticky note that read, *‘Thank you for sharing your story about ___. I had a similar experience during fellowship and it’s terrifying*.’ Others mentioned gratitude for the vulnerability of the storytellers, as in a sticky note that read, *‘To ___ – Your bravery in sharing your story is a gift. Thank you for giving voice to such a crushing experience. You helped me grieve along with you and our patients*.’ Participants also expressed gratitude for the event as a whole and voiced appreciation that medical education leadership was present and supportive. Please see Table 2 for examples of quotes demonstrating the theme *Gratitude*.

### Burnout

Most of the data specifically addressing burnout came from the follow-up questions. In response to, ‘*Do you think storytelling events like the Story Slam could help mitigate burnout? If so, how?’* a majority of respondents stated that storytelling events have potential to impact burnout. As one resident stated,

*I do think events like this help mitigate burnout, for the speaker, it is cathartic, for the audience it is inspiring and helps self-reflection*. [follow-up, resident #8]

The burnout theme contained 4 sub-themes, 3 of which affirmed the positive impact of the Story Slam: *Helpful for Impacting burnout, Helpful and needs to happen more often*, and *Helpful and systemic changes also needed*. The 4th sub-theme, *Unlikely to help*, was expressed by 2 respondents. Please see Table 3 for examples of quotes demonstrating this theme and sub-themes.

**Table 3.** Themes and sub-themes regarding potential impact of storytelling events on burnout

Theme	Sub-Theme	Example quotes
**Burnout**	Helpful for impacting burnout	Absolutely. I left that evening feeling connected, re-energized, and more empathetic towards my colleagues, especially those who are trainees, and their experiences. Anything that connects us to each other, our shared mission in medicine, and our humanity is protective against burn-out. [follow-up, faculty #1] I do think that storytelling events like this event can help burnout. I think we as a trainee [sic] who work in the medical field come across a variety of stress in both work and outside of work and sharing it, especially sharing the not so great experiences I think is a way to normalize the difficulty that we encounter every day. By normalizing this experience, I think it opens the dialogue we can express more compassion for each other as we understand each other more. [follow-up, resident #1] Yes! It seems a big factor in burn-out is the internal voice that tells physician trainees (and physicians) that they aren’t as good as their peers, that they are the only one struggling with the work, that they shouldn’t be a doctor, etc, etc. The story slam is a way for residents/physicians/students to see that we are all struggling with the same things, and that it helps to share our vulnerabilities with others. This is one of the few ways to breakdown the myth that everyone else is superhuman, never makes mistakes, never suffers from the work. [follow-up, faculty #4]
	Helpful and needs to happen more often	Yes, this can definitely help with burn out. However, I think the effect from the event would only last temporary, unless the culture of sharing story continues. I think this event encourages people to share their story, without feeling ashamed of guilt, or mistake which I [sic] will really help with the burnout. [follow-up, resident #2] Yes, absolutely. As providers in general, we often go to our peers to vent or talk through particularly difficult situations. Whether we are looking for reassurance that we made the right decision, or looking to hear someone say ‘that must have been so frustrating’ it is the people around us going through the same experiences that provide the most support. Even when our spouses, family members, or friends outside of medicine want to help, it is not the same level of understanding or support that those in the medical field can provide. This event is a way in which everyone who participates, whether passively or actively, feels a sense of community, and by extension, feels supported and validated in our feelings. This outlet will absolutely help mitigate burnout, but I think it would need to happen more regularly than once a year. [follow-up, resident #9] Yes, as like an instigating event, but something needs to happen to keep it going through time. And also by like the people you meet at the story event and the people that you hear, you then know you connected with them so you can reach out to them throughout the year, but that you need things like this to keep it going. By itself it can’t by itself mitigate burnout. [follow-up, resident #19 (storyteller)]
	Helpful and systemic changes also needed	I think in a way it can, by sharing stories and letting trainees know that they are not alone and giving a certain amount of validation to similar feelings that may be experienced. I think ultimately though the culture of medicine (duty hours, 24 hours calls, attending bullying, co-residents bullying, limited time to participate in one’s mental health due to other career demands) need to change too. [follow-up, fellow #] I think it is a bit like a flu shot-one will need booster shots to create some degree of protection. Similar to a flu shot, it can reduce the severity of burnout, and it’s not going to be sufficient or 100% effective on its own. Ultimately, we also need to create institutional policies and resources to make our training experiences more humane. [follow-up, faculty #]
	Unlikely to help	No, I think burnout is a result of a larger systems process that begins with the long, competitive, and expensive training process starting with MCATs and going through fellowships. Loved the event, but it will not make up for the 8+ years of delayed gratification, hundreds of thousands of dollars of debt, long work hours, living thousands of miles away from my family, cross cover pages, exhausting work days, low salary, poor diet & decreased exercise during busy months. [follow-up, resident #6] If you are depressed or burned out, one night of storytelling is not sufficient to make you feel better about systemic failures at work. When I was really depressed, events like this made me really angry. I felt like I was right to be angry and depressed and this was a system saying ‘don’t criticize us, just come to this event we are throwing and you will feel better’. But I do think storytelling events are a good occasion to gather with community, which is a positive coping mechanism for any suffering. [follow-up, resident #21 (storyteller)]

## Discussion

Our results support the use of storytelling events as a way to promote well-being in GME trainees by building connection and increasing resilience. Through our framework of social constructivism, these data clearly demonstrate that the Story Slam positively impacted participants, manifested as increased feelings of connection and community, a rekindling of meaning and purpose in their work, and a sense of gratitude. The telling of stories and the reflections on those stories were learning activities that helped foster well-being and resilience.

The identified themes tie to existing literature showing that feeling connected [17] and having a sense of meaning about work [18,19] promote resilience and decrease risk of burnout. In addition, gratitude has been shown to promote well-being and decrease burnout in health care professionals [17,43] which may serve as a mechanism through which storytelling events positively impact participants. Moreover, participants reported experiencing renewal and hope, and many believed the event had strong potential to mitigate burnout. As seen in quotes from participants, much of the power of the event was in hearing stories that resonated with their own experiences, serving as a reminder that they are surrounded by a community that can help them understand and learn from what they encounter in medicine. In the follow-up questions, many participants affirmed that the event prompted them to reflect on their own stories. This finding provides evidence for an instance of social constructivism, where individuals see a behavior modeled – in this case, reflecting on an experience in medicine – and then replicate the behavior.

Many participants mentioned being inspired by the vulnerability of the storytellers and described the storytellers as role models. This example of observational learning encourages us to consider how some participants may volunteer as storytellers at future events. In addition, some participants were inspired to use reflective practices themselves, suggesting that reflective practice is also a useful framework for understanding the mechanism through which storytelling events impact participants, regardless of whether they share a story.

Interestingly, the Story Slam’s positive impacts on fostering connection, meaning and hope, and gratitude were seen across the stages of medical training and practice, with many participants reporting positive impacts regardless of where they were in their career. Multiple residents commented that it was valuable to have faculty involved in the event, both as storytellers and audience members. Over the course of the evening and in Twitter exchanges afterward, residents and faculty were observed interacting and providing support to one another. Again, the lens of social constructivism helps us see that the shared experience – in this case across generations and career stages – is viewed positively by the participants. As Yoo and colleagues described, the opportunity to have intergenerational discussions and reflections may be an effective way to increase empathy and understanding and thus decrease burnout [44]. Just as social constructivism describes how modeling behaviors leads to learning those behaviors, this opportunity for intergenerational reflection also may help explain why the event was beneficial for faculty.

Our study has several limitations. First, although a large number of academic institutions and training programs were represented, all were located within a single upper Midwest metro area and had significant leadership support for this event. In a different geographical area or with less leadership support, participants’ experiences might differ from those reported here. Second, selection bias is possible since those enthusiastic about the Story Slam may be more likely to participate in the survey and agree to be interviewed. However, given our high response rates, we are confident that these themes describe the experience of a large portion of attendees. Third, we were unable to assess the duration of impact of the Story Slam, largely because the COVID-19 pandemic affected our ability to do long-term follow-up with attendees.

To address these limitations, we are exploring ways to collaborate with colleagues around the country to further study the impact of GME storytelling events. In addition, since COVID-19 inspired 2 virtual Story Slams for our Minnesota GME community, future research will explore whether virtual storytelling events have similar impacts as in-person gatherings. We also plan to evaluate the long-term impact of story-telling events in subsequent studies and may consider an approach to data analysis that would allow us to report the frequency with which themes arise in the data collected.

## Conclusions

Storytelling events in GME are well-received by faculty and trainees, can impact professional development through the frameworks of social constructivism and reflective practice, and are a promising tool for promoting resilience by building connection, rekindling a sense of purpose, cultivating gratitude, and instilling hope and renewal in attendees.

## Supplementary Material

Supplemental Material

## Prior publications

None of the data from this study have been presented elsewhere. We did publish an article describing the 2018 MMCGME Story Slam that served as a pilot study for this project: Olson ME, Walsh MM, Goepferd AK, Trappey B. Sharing Stories to Build Resilience: Articulating the Common Threads That Connect Us. *J Grad Med Educ*. 2019;11(3):340-341. doi:10.4300/JGME-D-18-00896.1
