# Supplementary material for: The strength of our stories: a qualitative analysis of a multi-institutional GME storytelling event
Source: Med Educ Online. 2021 Jun 7;26(1):1929798. doi: 10.1080/10872981.2021.1929798 (PMC8189054; doi:10.1080/10872981.2021.1929798)

Supplemental Digital Appendix 1. A sampling of sticky note reflections that participants wrote over the course of the evening.


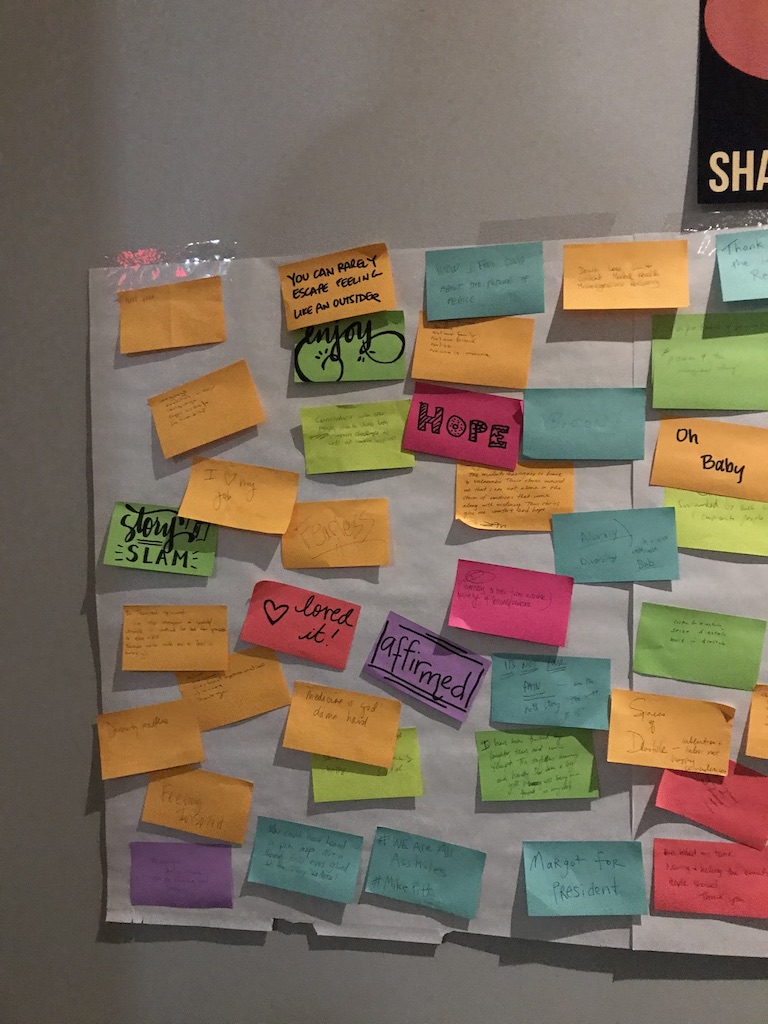


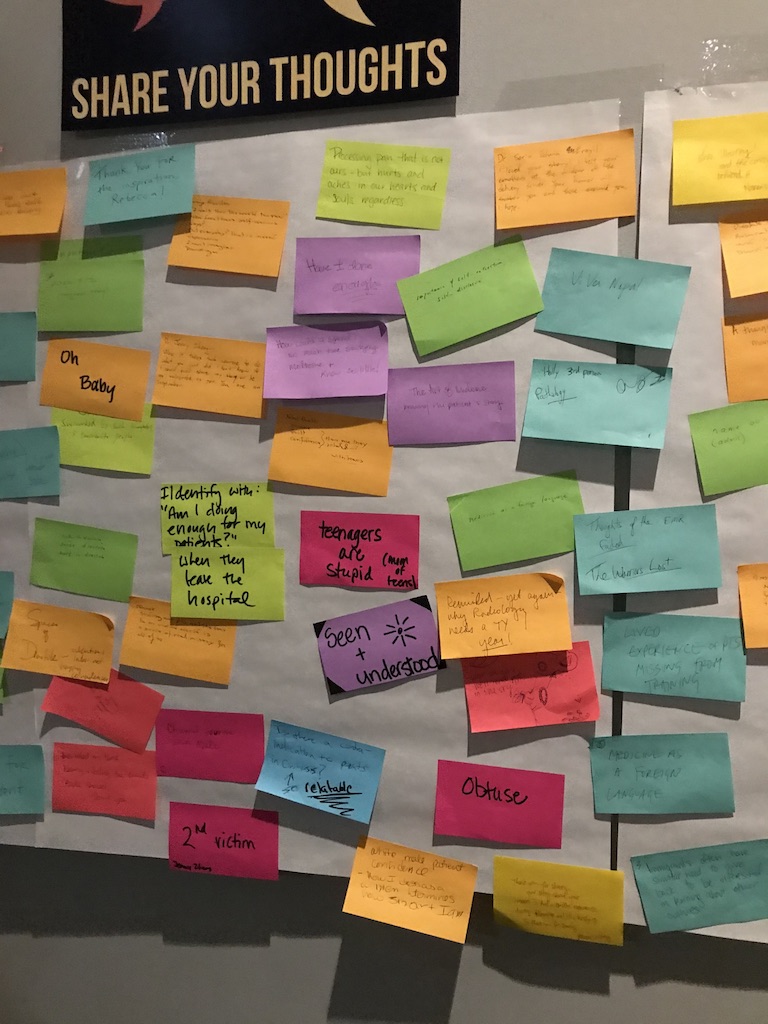


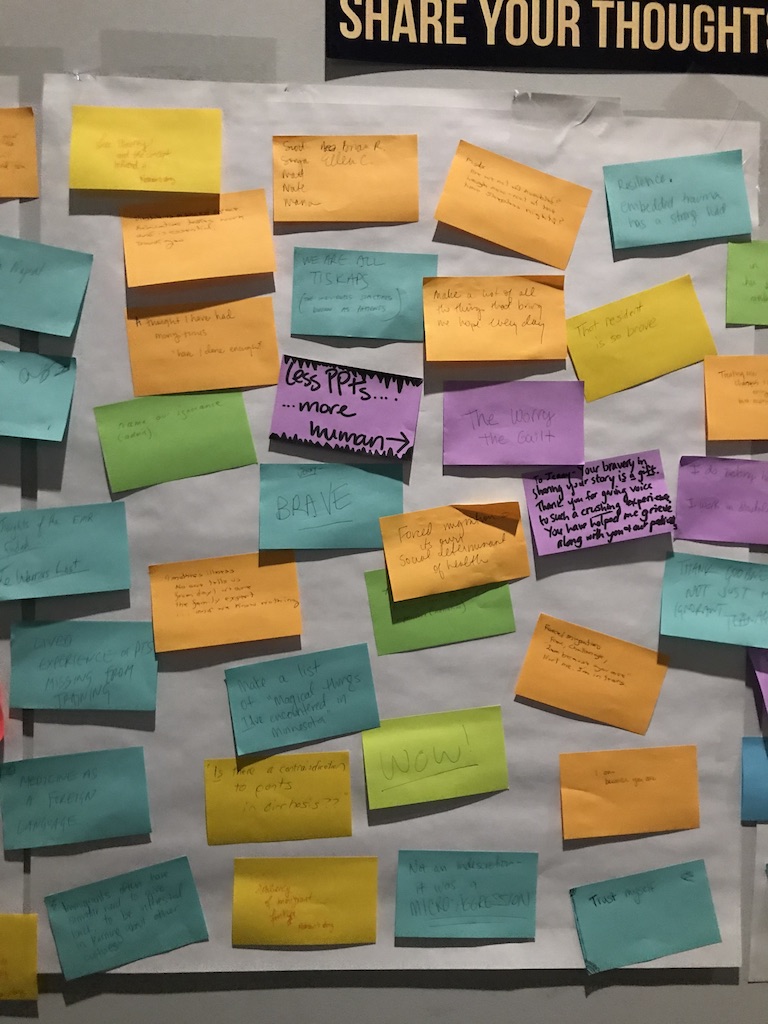

Supplement: Supplemental Material [file ZMEO_A_1929798_SM3918.zip › Supplementary files/Strength of Our Stories Supplemental Digital Appendix 1 .docx]
