# Supplementary material for: The strength of our stories: a qualitative analysis of a multi-institutional GME storytelling event
Source: Med Educ Online. 2021 Jun 7;26(1):1929798. doi: 10.1080/10872981.2021.1929798 (PMC8189054; doi:10.1080/10872981.2021.1929798)
Supplement: Supplemental Material [file ZMEO_A_1929798_SM3918.zip › Supplementary files/Strength of Our Stories Supplemental Digital Appendix 2_.docx]

**Supplemental Digital Appendix 2**

**Post-Event Survey for All Attendees**

1. Are you a:

- Resident
- Fellow
- Faculty member
- Other: ________ [fill in the blank]

1. Did you share a story tonight?

- Yes
- No

1. Describe your experience at tonight’s event (e.g. How did you feel? What surprised you? What was meaningful for you?) [text box]
2. One goal of the Story Slam was to help create a sense of connectedness among those who attended. Did the event accomplish that goal?

- Not at all
- A little bit
- Moderately well
- Quite well
- Extremely well

1. How likely would you be to recommend a future Story Slam to friends and colleagues?

- Not at all likely
- A little likely
- Moderately likely
- Quite likely
- Extremely likely

1. As part of a research project on the use of storytelling events in medical education, we are looking for people to participate in a brief interview about their experience at the Story Slam. Would you be willing to volunteer? Yes or no. [If yes, skip logic to get name & email.]
2. Any other comments or suggestions? [text box]
