# Supplementary material for: The strength of our stories: a qualitative analysis of a multi-institutional GME storytelling event
Source: Med Educ Online. 2021 Jun 7;26(1):1929798. doi: 10.1080/10872981.2021.1929798 (PMC8189054; doi:10.1080/10872981.2021.1929798)
Supplement: Supplemental Material [file ZMEO_A_1929798_SM3918.zip › Supplementary files/Strength of Our Stories Supplemental Digital Appendix 3.docx]

**Supplemental Digital Appendix 3**

**Interview Recruitment Email and In-Depth Interview Questions for all Attendees**

Hello!

Thanks so much for volunteering to spend a few minutes to share your perspective on the Story Slam. We really appreciate it!

In order to simplify things given everyone’s busy schedules, we’re offering 3 options for sharing your thoughts.

1. Record a voice memo (iPhone users), other audio message, or a video of yourself answering the questions below and send it to XXX via email.
2. Reply to this email with your answers to the questions.
3. Reply to this email to schedule a time for a phone or in-person interview with someone from our team.

Please pick the option that’s best for you and get back to us ASAP while the Story Slam is still fresh in your mind, ideally before Thanksgiving.  If you have any questions, please reach out.

Here are the questions we’d like you to answer:

- Are you a resident, fellow, attending, or do you have some other role?
- Do you think storytelling events like the Story Slam could help mitigate burnout? If so, how?
- At the close of the Story Slam, many people wrote about feeling connected or about feeling a sense of community. If you had either (or both) of those reactions, do you think it is likely to persist? Why or why not?
- After the Story Slam, have you been reflecting on any stories from your own experience? If so, why? Has your perspective on the experience changed after attending the Story Slam?
- How likely would you be to share a story at a storytelling event? Please explain
